# Impact of a multidisciplinary training programme on outcome of upper rectal cancer by critical appraisal of the extent of mesorectal excision with postoperative MRI

**DOI:** 10.1002/bjs5.50242

**Published:** 2019-12-13

**Authors:** P. Bondeven, S. Laurberg, R. H. Hagemann‐Madsen, B. G. Pedersen

**Affiliations:** ^1^ Department of Surgery Randers Regional Hospital Randers Denmark; ^2^ Department of Surgery Aalborg University Hospital Aalborg Denmark; ^3^ Department of Surgery Aarhus University Hospital Aarhus Denmark; ^4^ Department of Radiology, MR Research Centre Aarhus University Hospital Aarhus Denmark; ^5^ Department of Pathology Sygehus Lillebælt Vejle Denmark

## Abstract

**Background:**

Optimal management of patients with upper rectal cancer remains unclear. Partial mesorectal excision (PME) without neoadjuvant therapy is currently advocated for the majority of patients. Recent studies, however, reported a high risk of local recurrence and suboptimal surgery. The aim of this study was to evaluate the effects of a quality assurance initiative with postoperative MRI to improve outcomes in these patients.

**Methods:**

Patients who underwent mesorectal excision with curative intent for rectal cancer in 2007–2013 were included. Postoperative MRI of the pelvis was performed 1 year after surgery. In 2011, a multidisciplinary workshop with focus on extent and completeness of surgery was held for training surgeons, pathologists and radiologists involved in treatment planning. Images of residual mesorectum and histopathological reports were reviewed with regard to the distal resection margin. Local recurrence after a minimum of 3 years' follow‐up was compared between two cohorts from 2007–2010 and 2011–2013.

**Results:**

A total of 627 patients were included; postoperative MRI of the pelvis was done in 381 patients. The 3‐year actuarial local recurrence rate in patients with upper rectal cancer improved from 12·9 to 5·0 per cent (*P* = 0·012). After the workshop, fewer patients with cancer of the upper rectum were selected to have PME (90·8 per cent in 2007–2010 *versus* 80·2 per cent in 2011–2013; *P* = 0·023), and fewer patients who underwent PME had an insufficient distal resection margin (61·7 *versus* 31 per cent respectively; *P* < 0·001).

**Conclusion:**

Quality assessment of surgical practice may have a major impact on oncological outcome after surgery for upper rectal cancer.

## Graphical abstract

With a local quality assurance programme, significant improvement in local recurrence rates for cancer of the upper rectum was achieved by focusing on preoperative selection of type of surgery and the extent and completeness of mesorectal excision.

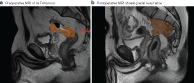

'Multidisciplinary training program helped to improve local recurrence rates'

## Abstract (es)

**Antecedentes:**

El tratamiento óptimo para los pacientes con cáncer del tercio superior de recto no está claro. En este momento, la conducta más empleada es la exéresis parcial del mesorrecto (*partial mesorectal excision*, PME) sin tratamiento neoadyuvante. Sin embargo, estudios recientes han apuntado que se trata de una cirugía subóptima con un elevado riesgo de recidiva local. El objetivo de este estudio fue evaluar los efectos de una iniciativa de control de calidad con una resonancia magnética (*magnetic resonance imaging*, MRI) postoperatoria para mejorar los resultados en estos pacientes.

**Métodos:**

Se incluyeron los pacientes con cáncer rectal a los que se realizó una exéresis del mesorrecto con intención curativa entre los años 2007 y 2013. Un año después de la cirugía se realizó una MRI de la pelvis. En el 2011, se organizó un taller multidisciplinario para educar a los cirujanos, patólogos y radiólogos involucrados en la planificación del tratamiento, en el que se discutieron la extensión y la radicalidad de la cirugía. Se revisaron las imágenes de mesorrecto residual y los informes histopatológicos respecto al margen de resección distal. Se comparó la recidiva local después de más de 3 años de seguimiento entre dos cohortes temporales, 2007‐2010 y 2011‐2013, respectivamente.

**Resultados:**

Se incluyeron un total de 627 pacientes, en los que en 381 se realizó una MRI postoperatoria de la pelvis. Las tasa actuarial de recidiva local a 3 años en pacientes con cáncer del tercio superior de recto mejoraron del 12,9% al 5,0% (*P* = 0,012). Después del taller, se realizaron menos PME en pacientes con cáncer del tercio superior de recto (91% *versus* 80%, *P* = 0,023) y menos pacientes en los que se realizó una PME presentaron un margen de resección distal insuficiente (62% *versus* 31%, *P* < 0,001).

**Conclusión:**

La evaluación de la calidad de la práctica quirúrgica puede tener un gran impacto en los resultados oncológicos después de la cirugía del cáncer del tercio superior de recto.

## Introduction

Management of rectal cancer has improved over recent decades, primarily by the standardization and improvement of surgical procedure. Whereas total mesorectal excision (TME) or abdominoperineal excision (APE) is optimal for mid and low rectal cancer, there has been little focus on the optimal management of upper rectal cancer. Tumours of the upper rectum (more than 10–15 cm from the anal verge) may not require TME, and may be managed optimally by perpendicular transection of the mesorectum at least 5 cm below the lower edge of the tumour. Partial mesorectal excision (PME) is currently advocated for the majority of tumours of the upper rectum, based on the rationale that preservation of a distal part of the rectum and mesorectum will result in better long‐term functional outcome and fewer postoperative complications, and assumed similar oncological safety^1^, ^2^, ^3^, ^4^, ^5^, ^6^. Dedicated centres have reported local recurrence rates of between 4 and 8 per cent following PME for upper rectal cancer, without the use of neoadjuvant treatment, equal to or better than the local recurrence rates for TME^2^, ^3^, ^7^, ^8^.

Standardization and quality assurance of mesorectal excision by training and pathological audit were implemented in the major trials to ensure that optimal surgery was performed. It is important, however, that outside the setting of clinical trials standardization and assurance of best quality surgery are also sustained in routine clinical practice. By using postoperative MRI it is possible to assess the extent and completeness of mesorectal excision by focusing on the tissue left behind, as opposed to tissue removed^9^, ^10^.

A concerningly high rate of local recurrence (13·5 per cent) in patients with upper rectal cancer after PME has been reported previously^11^. Evidence of suboptimal surgery and/or selection was clear^9^, ^11^. Similarly, in studies from Germany and Sweden local recurrence rates in patients with upper rectal cancer as high as 10–16 per cent have been reported^12^, ^13^, ^14^, ^15^. A difference of more than 10 per cent in the local recurrence rate most likely reflects variations in the preoperative assessment, surgical technique and/or use of neoadjuvant therapy.

The aim of this study was to assess the effect of a multidisciplinary‐initiated quality assurance programme in patients treated for upper rectal cancer with regard to local recurrence, type of surgery, residual mesorectum and distal resection margin.

## Methods

Consecutive patients with rectal adenocarcinoma, defined as cancer 15 cm or less from the anal verge on rigid proctoscopy, who underwent mesorectal excision with curative intent at Aarhus University Hospital or Regional Hospital Randers, two hospitals in Denmark, from August 2007 to 2013 were included in the study cohort. All patients were discussed at a multidisciplinary team (MDT) conference based on preoperative MRI of the pelvis (if applicable), CT of the chest and abdomen, and a clinical examination with rigid proctoscopy.

Danish guidelines^16^ recommend a selective approach in application of long‐course chemoradiotherapy for rectal cancer according to the stage and location of the tumour discussed at an MDT conference. All patients with resectable, cT1–2 upper, mid or low rectal cancer undergo surgery directly, without neoadjuvant treatment. Patients with a cT3 tumour of the mid rectum are allocated to long‐course preoperative radiochemotherapy if the distance from the tumour to the mesorectal fascia is less than 5 mm on preoperative MRI. cT3–4 tumours of the low rectum are all considered candidates for long‐course radiochemotherapy. In Denmark, only unresectable cancer of the upper rectum is considered for neoadjuvant therapy. Guidelines were unchanged during the study period.

Patients who had surgery without signs of distant metastasis and with tumours considered to be resectable with a clear margin at preoperative evaluation or after neoadjuvant therapy were classified as having treatment with curative intent, irrespective of the pathological circumferential resection margin (CRM) involvement at definitive histopathological examination. Type of surgery was determined from the surgical reports. Approval for the study was obtained from the Local Committee for Health Research Ethics and the Danish Data Protection Agency (2009‐41‐4056).

### Data collection and follow‐up

The length of the distal resection margin (DRM) was measured in all rectal cancer specimens at histopathological examination. The length of the DRM was measured on the fixed specimen after sectioning in 5‐mm slices, as the distance between the luminal lower border of the tumour and the distal cut edge. A DRM of less than 3·5 cm in PME resections was evaluated as insufficient based on findings of a previous study^17^.

Tumour height had been documented by both rigid proctoscopy at preoperative evaluation and on preoperative MRI. The height measured by rigid proctoscopy was set as the method of reference. On MRI, tumour height was measured as the distance between the lower border of the subcutaneous part of the external sphincter, reflecting the anal verge, and the most distal part of the luminal tumour.

Generally, patients were included in a follow‐up regimen with outpatient visits at 3, 6, 12, 18, 24 and 36 months after surgery. At each visit, a clinical examination and rigid proctoscopy (not applicable in patients who had APE) were performed. CT of the chest and abdomen was performed according to the protocol for the COLOFOL trial^18^ (NCT00225641) with frequent and less frequent arms. Follow‐up CT was performed at 12 and 36 months in the less frequent arm and at 6, 12, 18, 24 and 36 months in the frequent arm. A subset of elderly and co‐morbid patients were exempted from regular follow‐up and were not examined routinely for disease recurrence. Local recurrence was defined as a clinical, symptomatic, radiologically evident tumour or biopsy‐proven adenocarcinoma located in the pelvis, regardless of the presence of distant metastasis. Histological verification was not achieved in all patients and was not considered a prerequisite for diagnosis.

### Postoperative MRI

Consecutive patients were invited for postoperative MRI of the pelvis. Patients with disseminated disease, previous diagnosis of local recurrence, death at inclusion or contraindication to MRI were not eligible. Patients had MRI at least 6 months from the time of primary surgery.

MRI was performed using a Magnetom Avanto 1·5‐Tesla MRI Scanner® (Siemens, Erlangen, Germany). Sagittal, axial and coronal T2‐weighted turbo spin‐echo images were obtained in addition to a sagittal short T1‐inversion recovery of the bony pelvis and a sagittal T23D sequence of the smaller pelvis. The radiologist was blinded to the pathological assessment and all clinical data, with the exception of preoperative MRI findings and type of surgery. Evaluation of the postoperative MRI included assessment for the presence, localization and size of residual mesorectum, level of anastomosis and detection of local recurrence. The same radiologist evaluated all radiological examinations together with the first author for consensus. Mesorectal fatty tissue with a discernible tissue interface of fibrosis, which separates the mesorectum from the mesocolon, was considered a sign of residual mesorectum and categorized as described previously^9^. Only mesorectum above the level of the anastomosis perpendicular to the bowel was regarded as inadvertent residual mesorectum following PME.

### Intervention

Quality assessment of surgery in the two departments has been performed since 2007 by postoperative MRI and histopathological examination. In 2011, an MDT‐initiated workshop with focus on the extent and completeness of mesorectal excision was held with participation of all involved surgeons, pathologists and radiologists. A specific focus was on upper rectal cancer and PME based on postoperative MRI findings between 2007 and 2010. A description of the procedure with comparison of preoperative and postoperative images together with histopathological section was presented. Documentation of inadvertent residual mesorectum following mesorectal excision, length of DRM, histopathological examination, and comparison between tumour height on rigid proctoscopy and preoperative MRI were reviewed and discussed in a plenary session.

After the workshop, quality assessment of treatment and surgery continued by postoperative MRI and histopathological examination according to the same protocol.

### Statistical analysis

Demographic and patient characteristics were summarized using median (range) values and percentages, with comparisons performed using Student's *t* test and the χ^2^ test for continuous and categorical data respectively. Local recurrence rates were estimated using Kaplan–Meier actuarial methods between the two time periods, 2007–2010 and 2011–2013. The log rank test was used for comparisons, as well as for subanalysis in patients with tumours of the upper rectum. Time to local recurrence was measured from the date of primary surgery to the date of diagnosis of local recurrence. Patients without local recurrence were censored on the date of their last outpatient visit or upon death.

Statistical analyses were performed using Stata® version 12 (StataCorp, College Station, Texas, USA) and SigmaPlot® (Systat Software, San Jose, California, USA). *P* < 0·050 was considered to represent statistical significance.

## Results

A total of 627 patients underwent curative resection with mesorectal excision for rectal adenocarcinoma between 2007 and 2013. Clinical characteristics of these patients according to the two time periods are shown in *Table* 1. Distributions of age, sex, tumour stage and use of neoadjuvant therapy were similar between the two periods, 2007–2010 and 2011–2013, but more patients had laparoscopic surgery compared with open surgery in 2011–2013 (71·2 per cent *versus* 8·5 per cent in 2007–2010; *P* < 0·001). Of the 627 patients, 381 (60·6 per cent) had postoperative MRI performed a median of 12 months after primary surgery.

**Table 1:** Patient demographics, tumour and treatment characteristics

	2007–2010	2011–2013	*P* ‡
**No. of patients**	363	264	
**No. who had postoperative MRI**	212 (58·4)	169 (64·0)	0·160
**Age (years)** *	66 (31–92)	67 (40–91)	0·219§
**Sex**			0·742
M	218 (60·1)	156 (59·1)	
F	145 (39·9)	108 (40·9)	
**Distance of primary tumour from anal verge (cm)** †			0·878
> 10–15	141 (38·8)	101 (38·3)	
> 5–10	124 (34·2)	97 (36·7)	
0–5	97 (26·7)	66 (25·0)	
Missing	1 (0·3)	0 (0)	
**Preoperative radiotherapy**			0·462
No	264 (72·7)	199 (75·4)	
Yes	99 (27·3)	65 (24·6)	
**Type of surgery**			0·174
PME	128 (35·3)	81 (30·7)	
TME	126 (34·7)	111 (42·0)	
APE	109 (30·0)	72 (27·3)	
**Surgical approach**			< 0·001
Laparotomy	332 (91·5)	76 (28·8)	
Laparoscopy	31 (8·5)	188 (71·2)	
**pT category**			0·196
pT0–2	123 (33·9)	82 (31·1)	
pT3	195 (53·7)	159 (60·2)	
pT4	40 (11·0)	21 (8·0)	
Missing	5 (1·4)	2 (0·8)	
**pN category**			0·370
pN0	235 (64·7)	175 (66·3)	
pN1–2	120 (33·1)	87 (33·0)	
Missing	8 (2·2)	2 (0·8)	
**CRM**			0·150
Negative	316 (87·1)	238 (90·2)	
Positive	41 (11·3)	23 (8·7)	
Missing	6 (1·7)	3 (1·1)	
**Plane of surgery**			0·036
Muscularis propria	106 (29·2)	68 (25·8)	
Intrarectal or mesorectal	243 (66·9)	192 (72·7)	
Missing	14 (3·9)	4 (1·5)	

Values in parentheses are percentages unless indicated otherwise;

* values are median (range).

† Measured by rigid proctoscopy at preoperative clinical evaluation. PME, partial mesorectal excision; TME, total mesorectal excision; APE, abdominoperineal excision; CRM, circumferential resection margin.

‡ χ^2^ test, except

§ Student's *t* test.

After a median follow‐up of 36 (range 0·2–107) months, 42 patients developed local recurrence. The overall 3‐year actuarial local recurrence rates in 2007–2010 and 2011–2013 were 7·3 (95 per cent c.i. 4·9 to 10·9) and 4·6 (2·5 to 8·4) per cent respectively (*P* = 0·156) (*Table* 2). The risk of local recurrence was associated with advanced tumour stage and an involved CRM. Distant metastatic disease developed in 11·8 per cent, and did not differ between the two time periods (12·7 *versus* 10·6 per cent respectively; *P* = 0·454).

**Table 2:** Local recurrence rates according to treatment characteristics in the two cohorts

	3‐year actuarial local recurrence (%)		
	2007–2010	2011–2013	*P* *
**All patients**	7·3 (4·9, 10·9)	4·6 (2·5, 8·4)	0·156
**Sex**			
M	7·2 (4·3, 12·2)	4·7 (2·2, 10·3)	0·926
F	7·4 (2·8, 11·9)	5·0 (1·9, 12·6)	0·528
**Distance of primary tumour from anal verge (cm)**			
> 10–15	12·9 (8·0, 20·6)	5·0 (1·9, 12·9)	0·012
> 5–10	1·9 (0·5, 7·6)	3·9 (1·3, 11·5)	0·083
0–5	6·1 (2·6, 14·1)	5·9 (1·9, 17·1)	0·835
**Preoperative radiotherapy**			
No	8·5 (5·5, 13·1)	5·1 (2·6, 9·9)	0·681
Yes	3·9 (1·3, 11·4)	3·9 (1·0, 14·8)	0·936
**Type of surgery**			
PME	13·3 (8·1, 21·4)	6·3 (2·4, 15·8)	0·028
TME	2·9 (0·9, 8·6)	3·5 (1·1, 10·2)	0·159
APE	5·6 (2·3, 12·8)	5·4 (1·8, 15·7)	0·825
**Surgical approach**			
Laparotomy	7·3 (4·8, 11·1)	5·0 (1·6, 14·7)	0·842
Laparoscopy	7·7 (1·9, 27·4)	4·8 (2·3, 9·7)	0·819
**pT category**			
pT0–2	1·9 (0·5, 7·4)	1·6 (0·2, 10·7)	0·613
pT3	8·1 (4·8, 13·5)	3·9 (1·6, 9·2)	0·354
pT4	22·6 (11·5, 41·6)	25·8 (10·6, 54·9)	0·883
**CRM**			
Negative	6·1 (3·8, 9·7)	3·8 (1·8, 7·8)	0·576
Positive	19·1 (9·0, 37·6)	15·4 (5·2, 40·5)	0·993
**Plane of surgery**			
Muscularis propria	9·4 (4·8, 17·8)	6·7 (1·0, 14·0)	0·965
Intramesorectal	3·7 (1·2, 11·0)	5·6 (2·1, 14·1)	0·423
Mesorectal	8·2 (4·5, 14·7)	5·2 (1·9, 13·2)	0·420
**pN category**			
pN0	5·1 (2·8, 9·3)	4·4 (2·0, 9·6)	0·729
pN1	12·4 (6·4, 23·3)	9·6 (2·1, 17·0)	0·576
pN2	11·8 (5·0, 28·4)	9·5 (3·2, 26·7)	0·915

Values in parentheses are 95 per cent confidence intervals. PME, partial mesorectal excision; TME, total mesorectal excision; APE, abdominoperineal excision; CRM, circumferential resection margin.

* Log rank test.

**Figure 1:**
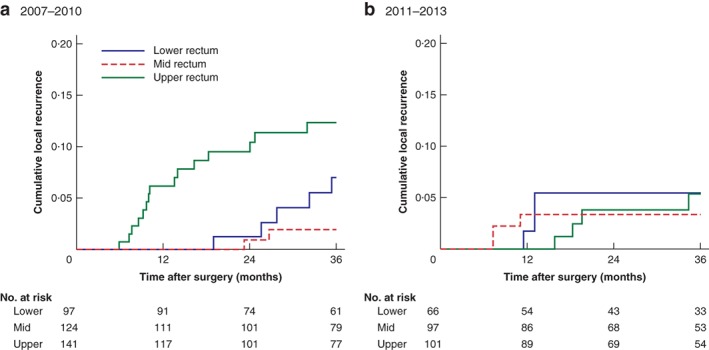
Actuarial local recurrence rates by tumour height in the two time periods **a** 2007–2010; **b** 2011–2013.

The risk of local recurrence in patients with cancer of the upper rectum improved after the workshop, from 12·9 per cent in 2007–2010 to 5·0 per cent in 2011–2013 (*P* = 0·012) (*Fig*. 1). Subanalysis of the risk factors for local recurrence in the group of patients with upper rectal cancer is shown in *Table* 3.

**Table 3:** Subanalysis of risk factors for local recurrence in patients with cancer of the upper rectum

	2007–2010		2011–2013		
	No. of patients (*n* = 141)	3‐year actuarial local recurrence (%)	No. of patients (*n* = 101)	3‐year actuarial local recurrence (%)	*P* *
**Type of surgery**					
PME	128 (90·8)	13·3	81 (80·2)	6·3	0·028
TME	13 (9·2)	0·0	20 (19·8)	0·0	–
**pT category**					
pT0–2	32 (22·7)	3·9	22 (21·8)	0·0	–
pT3	84 (59·6)	17·6	65 (64·4)	4·2	0·071
pT4	25 (17·7)	29·6	13 (12·9)	25·0	0·925
Missing	0 (0)	–	1 (1·0)	–	–
**CRM**					
Negative	128 (90·8)	11·5	91 (90·1)	4·2	0·081
Positive	13 (9·2)	28·6	9 (8·9)	13·3	0·834
Missing	0 (0)	–	1 (1·0)	–	–
**DRM pathology (only for PME)**	*n* = 128		*n* = 81		
< 3·5 cm	79 (61·7)	20·2	25 (31)	10	0·163
≥ 3·5 cm	43 (33·6)	0·0	55 (68)	0	–
Missing	6 (4·7)	–	1 (1)	–	–
**Surgical approach**					
Laparotomy	116 (82·3)	13·8	21 (20·8)	0·0	0·125
Laparoscopy	25 (17·7)	9·5	80 (79·2)	6·4	0·786

Values in parentheses are 95 per cent confidence intervals. PME, partial mesorectal excision; TME, total mesorectal excision; CRM, circumferential resection margin; DRM, distal resection margin.

* Log rank test.

**Figure 2:**
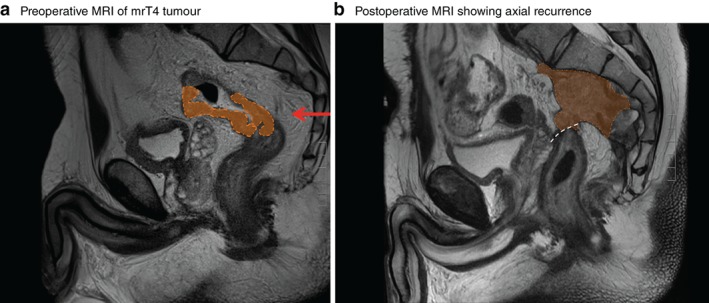
Sagittal T2‐weighted MRI of the pelvis **a** Preoperative MRI showing an mrT4 tumour (orange area) estimated to be located 8 cm from the anal verge on MRI, but which was measured by rigid proctoscopy before surgery to be located 12 cm from the anal verge. The red arrow denotes the height of tumour invasion. The patient was selected for partial mesorectal excision without preoperative radiochemotherapy. **b** Postoperative MRI 6 months after primary surgery in 2009, showing axial recurrence (orange) at the anastomosis (white dashed line). Suboptimal surgery with a distal resection margin of 8 mm was found on histopathological examination.

**Figure 3:**
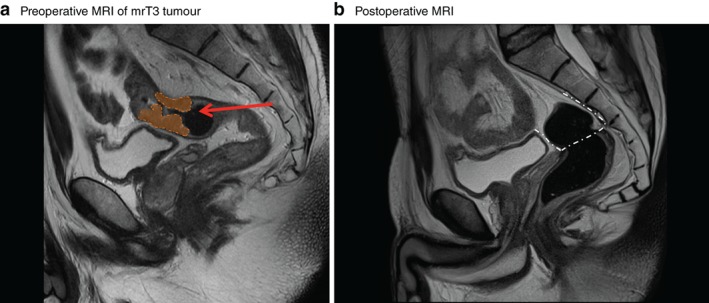
Sagittal T2‐weighted MRI of the pelvis **a** Preoperative MRI showing an mrT3 tumour located 14·8 cm from the anal verge in the upper rectum (orange area). The red arrow denotes the height of tumour invasion. **b** Postoperative MRI shows the plane of dissection and level of anastomosis (white dashed line). Only mesorectum below the level of the anastomosis is observed in a distance of more than 3·5 cm from the primary tumour.

Tumour location with regard to tumour height measured by rigid proctoscopy did not differ between 2007–2010 and 2011–2013, with 38·8 and 38·3 per cent respectively of tumours located in the upper rectum (*P* = 0·878) (*Table* 1). All patients who underwent PME had a tumour located more than 10 cm from the anal verge as measured by rigid proctoscopy. Of patients who had PME in 2007–2010, 22·7 per cent of the tumours (29 of 128) were estimated to be located in the mid rectum (5–10 cm from the anal verge) on preoperative MRI (*Figs* 2 and 3). This decreased to 5 per cent (4 of 81) in patients who underwent PME in 2011–2013 (*P* = 0·024). After the workshop, fewer patients with upper rectal cancer underwent PME compared with TME; the rate of TME increased from 9·2 per cent in 2007–2010 to 19·8 per cent in 2011–2013 (*P* = 0·023). No patient who had TME for cancer of the upper rectum developed local recurrence (*Table* 3).

Of all patients who underwent PME, the DRM measured at histopathological examination was reported to be less than 3·5 cm in 49·8 per cent (104 of 209). This improved in the later cohort, with a decrease from 61·7 per cent in 2007–2010 to 31 per cent in 2011–2013 (*P* < 0·001). No patient treated with PME developed local recurrence when a DRM of at least 3·5 cm was achieved (*Table* 3).

Inadvertent residual mesorectum was identified in 163 (42·8 per cent) of the 381 patients who had postoperative MRI. In patients who underwent PME, inadvertent residual mesorectum was detected in 60·4 per cent (84 of 139). This did not differ between 2007–2010 and 2011–2013: 60 per cent (50 of 84) and 62 per cent (34 of 55) respectively (*P* = 0·458). The mean volume of inadvertent residual mesorectum was 8·03 and 5·74 cm^3^ in 2007–2010 and 2011–2013 respectively (*P* = 0·250).

Based on the preoperative tumour status (mrT), T4 disease was overrepresented in patients with local recurrence who underwent PME or APE. All pT4 tumours of the upper rectum were due to local peritoneal involvement (pT4a).

In 2007–2010 and 2011–2013, long‐course preoperative radiochemotherapy was administered in 24·6 per cent (31 of 126) and 22·5 per cent (25 of 111) respectively of patients who had TME, and in 62·4 per cent (68 of 109) and 51 per cent (37 of 72) of those who underwent APE. In patients who had PME, neoadjuvant therapy was not used in 2007–2010, whereas it was employed in 4 per cent (3 of 81) of patients who underwent PME in 2011–2013.

## Discussion

This study has shown a significant reduction in the risk of local recurrence after a local MDT‐directed quality assurance programme in patients with cancer of the upper rectum. A previous report^11^ on local recurrence in patients who had PME for upper rectal cancer was disappointing, and suboptimal surgery and/or selection was evident.

To obtain and maintain improvements in quality of rectal cancer surgery, it may be necessary to consolidate the training by organizing continuous workshops with a focus on difficulties in the current treatment of rectal cancer, in order to sustain best quality treatment in routine clinical practice. By using postoperative MRI, it is possible to assess the extent and completeness of mesorectal excision, to highlight difficulties in current management of upper rectal cancer. One of the great hallmarks of MDT collaboration is quality assurance and internal audit of the quality of treatment performed.

For cancer of the upper rectum, it remains unsettled as to whether TME is necessary, or whether PME is adequate. Unfortunately, the major studies investigating the initial improvements following the introduction of TME have not specifically distinguished the type of procedure for upper rectal cancer, hindering comparison^19^. It is clear, however, that the less extensive PME with preservation of a distal part of remnant rectum offers a better functional outcome and lower risk of anastomotic leakage^1^, ^2^, ^20^. Dedicated single centres have shown that with skilful surgery it is possible with PME to achieve low local recurrence rates, equal to or better than the local recurrence rates achieved with TME, and without the use of neoadjuvant therapy^2^, ^3^, ^7^, ^8^.

Based on data from the Stockholm Colorectal Cancer Study Group, Syk and colleagues^21^, ^22^ reported a crude local recurrence rate of 9 per cent in patients who underwent PME for upper rectal cancer, despite the wide use of preoperative short‐course radiotherapy. Rosenberg and co‐workers^12^ performed PME for all tumours of the upper rectum and observed a 5‐year local recurrence rate of 15·5 per cent, not significantly different from that for mid‐rectal tumours, but worse than for sigmoid cancer. Similarly, Kodeda *et al*.^13^ reported a crude 5‐year local recurrence rate of 14·4 per cent for tumours of the upper rectum without routine preoperative radiotherapy in their regional cohort, compared with a local recurrence rate of 5·5 per cent from the Swedish Rectal Cancer Registry.

The risk of local recurrence in patients with upper rectal cancer treated in 2007–2010 corresponds to the results presented by Syk and colleagues^22^, in which CT and MRI images from 99 patients with local recurrence were analysed. In the group of patients with local recurrence, residual mesorectum was observed in 86 per cent on postoperative MRI. The authors suggested that an intentional or inadvertent PME, combined with the omission of radiotherapy, was the main cause of recurrence in these patients.

An effort to detect suboptimal surgery with postoperative imaging was attempted before the advent of modern MRI. By using postoperative angiography of the mesenteric artery, Hohenberger and co‐workers^23^ observed that the inferior mesenteric artery and superior rectal artery remained together with adjacent mesorectal fatty tissue after coning of the mesorectum.

In a recent educational tutorial by Kuzu *et al*.^24^, Heald stated that during a PME the rectum and mesorectum should be mobilized at least 8 cm below the level of the tumour to achieve an adequate margin in the fresh specimen of at least 5 cm. The fact that no patient who underwent PME with a distal margin greater than 3·5 cm developed local recurrence, compared with 17·7 per cent of patients with a shorter distal margin, further supports this recommendation. In 2012, Jullumstrø and colleagues^25^ analysed 394 patients in a Norwegian cohort with regard to violation of treatment guidelines. The risk of local recurrence was 11 per cent after curative resection with a distal clearance of less than 2 cm, compared with only 3 per cent when distal clearance was more than 2 cm.

Kusters and co‐workers^19^ analysed the patients with local recurrence in the Dutch TME Trial and found that most local recurrences were located in relation to the anastomosis, especially in the patients without the otherwise greatest risk factors (T4 tumour, N2 disease and involved CRM). Local recurrence in relation to the anastomosis is generally considered to be the result of inadequate or suboptimal surgery, with failure of complete surgical removal of the primary tumour or its initial field of spread in the mesorectum^19^, ^26^, ^27^, ^28^.

No difference in the reported prevalence of inadvertent residual mesorectum on postoperative MRI was observed in the present study after the workshop, despite the observed improvement in local recurrence. Small amounts of residual mesorectum in sufficient clearance of the primary tumour may not be associated with an increased risk of local recurrence, similar to pathology reports that an intramesorectal excision may not be associated with an increased risk of local recurrence compared with that following complete mesorectal excision^29^, ^30^.

There is ongoing controversy as to whether patients with cancer of the upper rectum will benefit from neoadjuvant therapy. In Denmark, only patients with unresectable cancer of the upper rectum are considered for neoadjuvant therapy, as opposed to those with advanced mid or low rectal cancer. Large population‐based studies from Sweden have reported that more than 60 per cent of patients with rectal cancer received neoadjuvant therapy, including 43–58 per cent of patients with tumours located in the upper rectum, and more than 40 per cent of those with T1–2 tumours^15^, ^31^. In 2011, Tiefenthal *et al*.^15^ showed an effect on local recurrence risk with preoperative short‐course radiotherapy for upper rectal tumours in a national population‐based study. Advanced tumours of the upper rectum can be staged accurately using MRI and also possibly downstaged with neoadjuvant therapy^32^, ^33^.

The characterization of rectal cancer into high, mid and low lesions is traditionally measured from the anal verge using a rigid proctoscope. Although this measurement is mandatory in Denmark, it may vary depending on the surgeon performing the examination, the patient and the method^34^. Confident localization of a rectal tumour is increasingly important, especially in relation to treatment guidelines, as the differentiation between mid or upper rectum has a significant impact on the decision to allocate for neoadjuvant therapy.

In their 2016 annual report^35^ on colorectal cancer management in Denmark, the Danish Colorectal Cancer Group reported that 20 per cent of 377 tumours located in the upper rectum, when measured by rigid proctoscopy, were found to be located in the mid rectum on preoperative MRI. The English National Low Rectal Cancer Development Programme^36^ has suggested a new definition of low rectal cancer as a tumour with its distal margin at or below the level of origin of the levators on the pelvic side‐wall. In the same way, because division of the rectum by strict centimetre criteria seems unrealistic on an individual basis, tumours of the upper rectum were defined by anatomical landmarks appreciable on preoperative MRI, such as the level of the peritoneal reflection or length of the mesorectum to the pelvic floor^37^, ^38^. By relying more on preoperative MRI to determine the location of the tumour in the rectum, fewer patients were selected to undergo PME. In advanced cases there may be an increased risk when leaving behind mesorectum. Similar findings were reported by Chang and colleagues^39^ in a study that used MRI to identify high‐risk patients with upper rectal cancer.

In the present authors' opinion, the height of the tumour is assessed most reliably by preoperative MRI, and can be used to determine whether PME or TME is preferable. By using the Delphi method, the European Registration of Cancer Care^40^ has recommended MRI as the method of choice for assessing tumour height and location in the rectum in multidisciplinary treatment of rectal cancer.

Limitations of the present study include the differences in the baseline characteristics between the patient cohorts in the two time periods, and that the implementation of laparoscopic surgery in the second period may also have potentially influenced outcomes. All MRI examinations were evaluated by the same radiologist in consensus with the first author. No previous analyses on interobserver variability have been reported. In a recent study by Veltcamp Helbach *et al*.^10^, two radiologists evaluated the MRI images independently. After the first evaluation, there was agreement of the radiologists in 59·4 per cent of cases. After consensus reading, consensus was reached in all patients as the radiologists became more familiar with identifying residual mesorectum on MRI, which implicates a learning curve. In the present study, residual mesorectum was considered to be present only when consensus was achieved.

A national audit of the quality of surgery in Denmark is currently being conducted including postoperative MRI, and a training programme for colorectal surgeons with a specific focus on surgery for upper rectal cancer is planned.
